# Characterization of the honeybee AmNa_V_1 channel and tools to assess the toxicity of insecticides

**DOI:** 10.1038/srep12475

**Published:** 2015-07-23

**Authors:** Pascal Gosselin-Badaroudine, Adrien Moreau, Lucie Delemotte, Thierry Cens, Claude Collet, Matthieu Rousset, Pierre Charnet, Michael L. Klein, Mohamed Chahine

**Affiliations:** 1 Centre de recherche, Institut universitaire en santé mentale de Québec, Quebec City, QC, G1J 2G3 Canada; 2 Institute of Computational Molecular Science, Temple University, Philadelphia, PA 19122 USA; 3 Department of Medicine, Université Laval, Quebec City, QC, G1K 7P4 Canada; 4 Centre de Recherche de Biochimie Macromoléculaire (CRBM), CNRS UMR 5237, 1919 Montpellier, France; 5 INRA UR 406, Abeilles et Environnement, Domaine Saint Paul - Site Agroparc, CS40509, 84914 Avignon Cedex 9, France

## Abstract

Pollination is important for both agriculture and biodiversity. For a significant number of plants, this process is highly, and sometimes exclusively, dependent on the pollination activity of honeybees. The large numbers of honeybee colony losses reported in recent years have been attributed to colony collapse disorder. Various hypotheses, including pesticide overuse, have been suggested to explain the disorder. Using the *Xenopus* oocytes expression system and two microelectrode voltage-clamp, we report the functional expression and the molecular, biophysical, and pharmacological characterization of the western honeybee’s sodium channel (*Apis Mellifera* Na_V_1). The Na_V_1 channel is the primary target for pyrethroid insecticides in insect pests. We further report that the honeybee’s channel is also sensitive to permethrin and fenvalerate, respectively type I and type II pyrethroid insecticides. Molecular docking of these insecticides revealed a binding site that is similar to sites previously identified in other insects. We describe *in vitro* and *in silico* tools that can be used to test chemical compounds. Our findings could be used to assess the risks that current and next generation pesticides pose to honeybee populations.

The pollination of various plants by bees is essential for biodiversity as well as for agriculture. The value of pollination services in the United States was estimated at US $14.6 billion in 2000^1^. A more recent estimation has put the value of these services at approximately US $205 billion a year worldwide^2^. Despite the immense importance of honeybees for agriculture, reports of unusual colony losses continue to come in from the United States^3^ and elsewhere around the world^4^. These colony losses, which are characterized by the disappearance of adult bees from hives as well as abandoned food and brood, have been attributed to colony collapse disorder (CCD)^5^

Various hypotheses have been put forward to explain the cause of CCD, including insect-borne bacterial and fungal diseases, overuse of in-hive chemicals, overuse of agricultural pesticides, and use of genetically modified crops^5^. Bacterial and fungal diseases can be ruled out as they preferentially affect larvae and pupae whereas CCD is characterized by the disappearance of adult bees^6^,^7^. The overuse of commonly used insecticides and the introduction of new chemicals thus appear to be the most reasonable explanation for CCD given that such practices may occur in the vicinity of large beekeeping operations.

While both the lethal and sub-lethal effects of neonicotinoid insecticides on honeybees have been the focus of many investigations recently (see Goulson 2013 for a review)^8^, few studies have focused on pyrethroid insecticides, which are the second most commonly used type of insecticide in agriculture today^9^ and are also widely used in combatting malaria and other insect-borne diseases^10^.

Pyrethroid insecticides target voltage gated sodium channels in mammals and insects^11^,^12^,^13^. Based on their homology with known sodium channels, two genes were identified as putative sodium channels in the *Drosophila* proteome, *para* and *DSC1*^14^. Homologs of these proteins have also been predicted based on nucleotide sequences obtained from the *Apis mellifera* genome^15^.

Para stands for paralytic sodium channel because it was identified by locating the locus of mutations that induce temperature-sensitive paralysis phenotypes^16^. *DSC1* stands for *Drosophila* sodium channel 1. The gene for this *Drosophila* channel was isolated based on its homology with a mammalian sodium channel DNA probe^17^. Recent experiments indicate that, while this channel resembles a sodium channel, the protein would be mainly permeable to calcium^18^,^19^. However, the para channel’s sequence and function would be more similar to known Na_V_ channels. Thus, recent literature refers to this channel as Na_V_1. Na_V_1 is known as the principal target for sodium channel-specific pyrethroid insecticides^20^. In addition, mutations linked to knockdown resistance to pyrethroids have only been reported in the corresponding gene in other insects^21^ and in a gene coding for an enzyme that metabolizes such compounds^22^. In *Drosophila*, this channel is associated with a TipE and TipE homolog (TEH) regulatory subunit^23^,^24^. The original TipE subunit was named based on the location and phenotype induced by mutations in *Drosophila* (temperature-induced paralysis phenotype, locus E)^25^.

Sodium channels are responsible for the initiation of the action potential in many excitable cells. In mammals, they are essential for muscle contraction^26^, heart rhythmicity^27^, and neuronal firing^28^. However, in the presence of pyrethroid insecticides, the sodium channel deactivation and inactivation processes are disrupted^29^ leading to abnormal electrical activity, which has also been observed in honeybee antennal olfactory receptor neurons^30^. Moreover, sub-lethal doses have been shown to impair honeybee learning and memory^31^.

Mammalian sodium channels all feature four homologous domains^32^, each of which is composed of six transmembrane segments (S1–S6). The S1 to S4 segments of each domain form the voltage sensitive domain (VSD), which is responsible for driving the conformational changes of the protein. Upon depolarization, the VSDs activate, leading to the opening of the pore formed by the assembly of S5 and S6 of all the domains. Sodium ions then permeate and induce an inward current corresponding to the rising phase of the action potential.

Mammalian sodium channels are often co-expressed with one or more regulatory subunits (β_1–4_) whose function is to modulate the activity of the channels and increase expression levels^33^. The regulatory subunits for the Na_V_1 insect channels seem to have the same functions. However, the TipE and TipE homologous subunits (TEH) are quite different from the β_1–4_ subunits since they appear to feature two transmembrane segments^24^.

The functional characterization and molecular modeling of the *Apis mellifera* honeybee Na_V_1 channel (AmNa_V_1) reported here provides *in vitro* and *in silico* tools for assessing the toxicity of chemical compounds for honeybees. This could be an important step toward identifying the causes of CCD and designing new pesticides that are less toxic for honeybees.

## Results

### Sequence and molecular features of the AmNa_V_1 channel

A protein blast search revealed that the sodium channel sequence in the honeybee, as expected, features four homologous domains, which is characteristic of mammalian sodium and calcium channels. A protein profile analysis using hidden Markov models (HMMs) indicated that the AmNa_V_1 channel has a protein profile similar to the mammalian sodium and calcium channels. The hmmsearch algorithm using a protein profile inferred from manually curated sequences of mammalian sodium and calcium channels on the whole honeybee proteome as a query yielded a significant alignment with the AmNa_V_1 channel (e-value of 8.9E−24).

**Figure 1:**
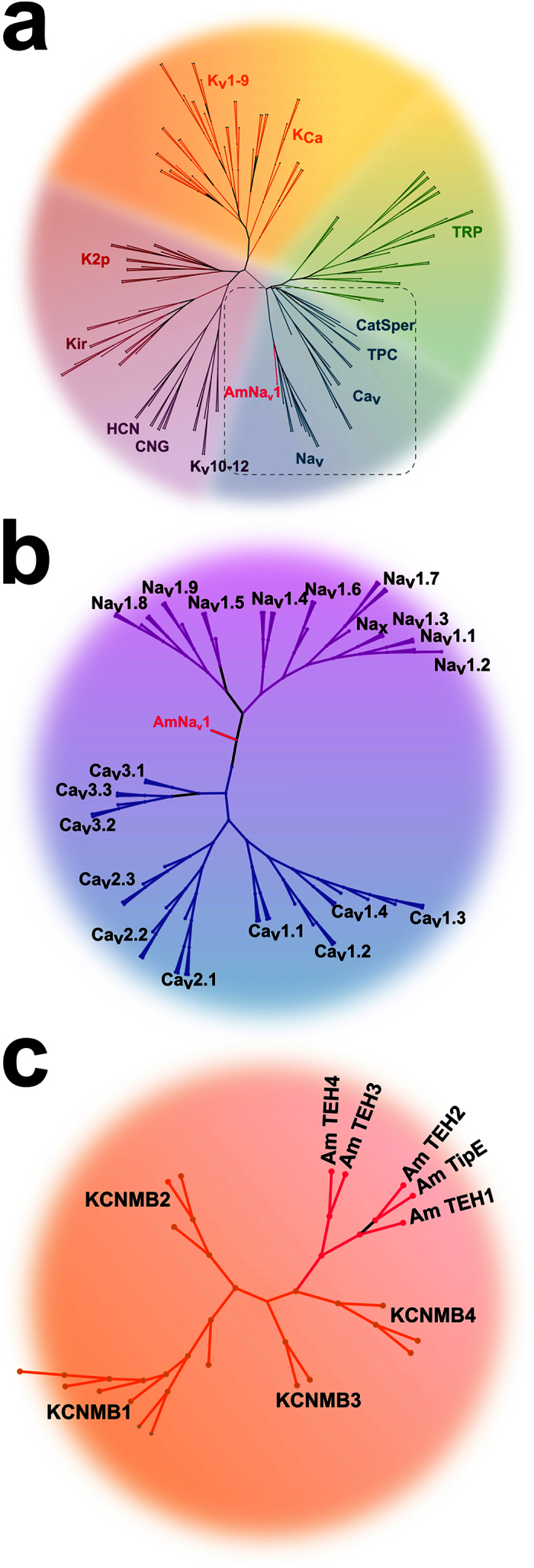
Phylogenetic classification of the AmNa_V_1 channel and its regulatory subunits. The minimum pore sequence of the VGL-chanome protein was aligned with the minimum pore sequence of the AmNa_V_1 channel. **(a)** Consensus phylogenic tree generated with the maximum likelihood method (using PROML of the PHYLIP package) show that the pore of the AmNa_V_1 channel diverged in its evolution from the mammalian sodium channels. **(b)** The tree generated by an alignment of the whole channel with all manually curated sequences of the mammalian sodium and calcium channels shows that the whole sequence diverged early in its evolution. **(c)** The regulatory subunits of the AmNa_V_1 channel yielded a significant alignment with *KCNMB* family members (regulatory subunits of mammalian K_Ca_ channels). The resulting tree shows that the TEH family diverged from the *KCNMB* family members, establishing a family of its own. Based on the protein sequences, the TEH family can be divided in two subfamilies.

A comparison of the AmNa_V_1 channel minimum pore sequence as defined by Yu and Catterall^34^ to members of the voltage gated-like chanome (VGL-chanome) showed that the pore of the honeybee channel arose from the same ancestor as mammalian sodium channels (Fig. 1a–b). This comparison of the whole protein with mammalian sodium and calcium channels indicates that the honeybee Na_V_1 channel is a homolog of mammalian sodium channels which would have branched away from these channels early in evolution (Fig. 1c).

Five protein sequences homologous to the *Drosophila* melanogaster Na_V_1 channel regulatory subunits were found in the honeybee proteome. These proteins appeared to share a common ancestor with mammalian K_Ca_β subunits (*KCNMB*) (Fig. 1d). Further information on the protein sequences of these regulatory subunits can be found in the Supplemental Information section.

A sequence alignment of the four homologous domains with the domains of other voltage gated sodium channels showed that the honeybee Na_V_1 channel, like that of *Drosophila*, shares many features with mammalian sodium channels (Figure S1). Since it fits the protein profile of mammalian sodium and calcium channels, each domain of the Na_V_1 channel most likely features six transmembrane segments. The conserved residues highlighted in S1 to S3 show that the amino acids that form the VSD gating charge transfer center (GCTC)^35^ are completely conserved in each domain. Furthermore, the positively charged residues on the S4 segment that interact with the GCTC are also fully conserved, indicating that the topology of each VSD is similar to the topology described in our previously published model of the human Na_V_1.4^36^.

The highlighted residues between the S5 and S6 segments in Figure S1 show that the selectivity filter of the AmNa_V_1 sodium channel is similar to that of mammalian sodium channels where two clusters of residues delimiting the ion conducting pore determine channel selectivity and sensitivity to tetrodotoxin (TTX)^37^,^38^. The clusters each consist of four conserved amino acids located in the re-entrant loop of the pore (DEKA and EEDD). In the honeybee Na_V_1 channel, the DEKA cluster is conserved while the EEDD cluster is substituted by EEQD (Figure S1a).

Alignments of the whole sequence indicated that the *Apis mellifera* Na_V_1 channel, like mammalian sodium channels, features a cluster of hydrophobic residues in the intracellular loop that links domains III and IV. In mammals, this cluster is involved in fast inactivation^39^. However, in insect channels, the MFMT sequence forms the inactivation particle. This sequence is similar to the IFMT sequence in mammalian channels (Figure S1b). The protein blast query also identified a putative site (the highly conserved serine S1581) for phosphorylation by PKC immediately after the hydrophobic cluster. (Figure S1b–c).

Alternative sequences for the Na_V_1 channel attributable to splice variants were also uncovered during our investigation (Figure S2a). They are the result of insertions or deletions of 9 to 11 residues in the large intracellular loops (DI/DII, DII/DIII, and DIII/DIV linkers). The large intracellular loops of mammalian sodium channels are typically associated with phosphorylation sites^32^ and protein-protein interaction sites^40^,^41^. No RNA editing sites were found in the Na_V_1 sequence.

We did however find sequence variations for the TipE and TEH4 regulatory subunits which were attributable to RNA editing and genomic variations (Figure S2b). Indeed, three single amino acid substitutions encoded in the cDNA produced from honeybee heads were also found in the bee’s genomic DNA for TipE. One such substitution was found for TEH4. Those sequence variants were classified as genomic variations. Two single nucleotide substitutions coding for a change in the amino acid sequence of TipE were found in our cDNA banks. Since the corresponding variations could not be found in the bees’ genomic DNA, those were attributed to RNA edition.

### Analysis of the AmNa_V_1 channel and the regulation of its regulatory subunits

**Figure 2:**
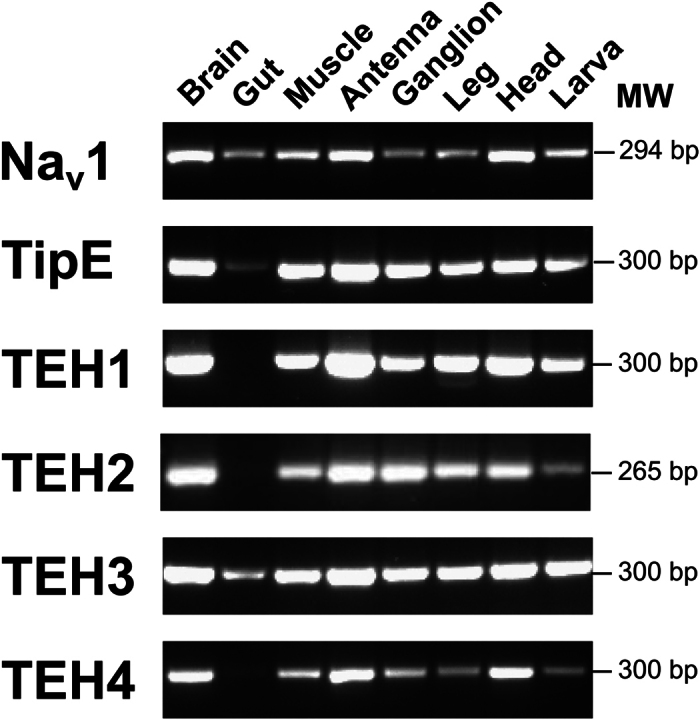
Expression of the AmNa_V_1 channel and its regulatory subunits in honeybee tissues. The tissue-specific expression of proteins was assessed by RT-PCR. All the honeybee tissue samples underwent the same preparation steps from dissection to gel electrophoresis. The expected weights of the amplicons are displayed in base pairs (bp).

An RT-PCR analysis showed that RNA corresponding to the AmNa_V_1 channel is expressed in all the honeybee tissues studied (brain, antenna, muscle, legs, gut, and ganglion). AmNa_V_1 channel’s RNA was also present in the larvae (Fig. 2). This suggests that the AmNa_V_1 channel plays a major role in the physiological functions of the honeybee.

The regulatory subunits of the AmNa_V_1 channel were also widely expressed in these tissues, with the exception of TEH1, which was not expressed in the gut. TEH2 and TEH4 also appeared to be expressed at very low levels in the gut (Fig. 2).

### Biophysical characterization

#### The regulatory subunits modulate the expression of the AmNa_V_1 channel

**Figure 3:**
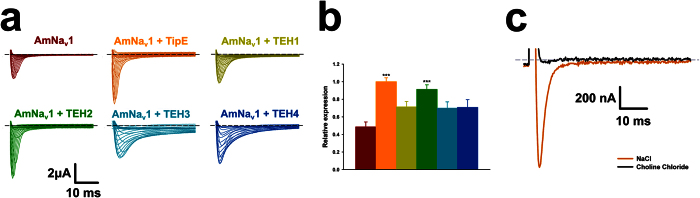
Effect of the co-expression of the regulatory subunits on the expression of AmNa_V_1. (**a**) Representative current traces from the pulse protocols applied to oocytes expressing either AmNa_V_1 alone or AmNa_V_1 and one of its regulatory subunits. The pulse protocols consisted of imposing−80 mV to +40 mV voltage steps in 5 mV increments. The oocytes were kept at the holding potential (−80 mV) for 1 s between voltage steps. (**b**) Relative amplitudes of the peak currents from oocytes expressing the AmNa_V_1 alone or with one regulatory subunit. The peak currents from 13–14 oocytes in response to a −80mV to −20 mV voltage step were measured for each bar. The mean was then normalized to the mean current amplitude measured in the presence of the TipE subunit. The differences between the means were then evaluated for statistical significance using a t-test with Bonferroni’s correction (***significant difference with AmNa_V_1 alone, *p* < 0.001). The oocytes came from the same batch and were tested the same day. (**c**) Representative current traces of an oocyte expressing AmNa_V_1 and TipE in response to a −80 mV to −20 mV voltage step. The current trace in orange was acquired in normal Ringer’s solution while the current trace in black was acquired in Ringer’s solution in which the sodium was replaced by choline.

The co-expression of the regulatory subunits with the AmNa_V_1 channel was not required to record sodium currents in *Xenopus* oocytes, indicating that it is robustly expressed on the membrane (Fig. 3a). Raw data from standard pulse protocols showed that the honeybee TipE and TEH subunits affect the kinetics of the channel (Fig. 3a). In addition, the co-expression of the honeybee TipE and TEH subunits with the Na_V_1 channel increased the peak current (Fig. 3b). However, only the co-expression of the Na_V_1 channel with the TipE or TEH2 subunits yielded a statistically significant increase in expression levels.

As indicated by the sequence analysis, the AmNa_V_1 channel is selective for sodium ions. Indeed, removing sodium ions from the extracellular medium abolished the current produced by the AmNa_V_1 channel (Fig. 3c).

#### Voltage-dependence of activation of the AmNa_V_1 channel

Like its mammalian counterparts, the AmNa_V_1 channel is activated by membrane depolarizations. The conductance-voltage relationship (G–V curve) of the channel fits a Boltzmann function with a V_1/2_ of −31.7 ± 0.9 mV and a slope factor (k) of –2.9 ± 0.2 mV (n = 11).

**Figure 4:**
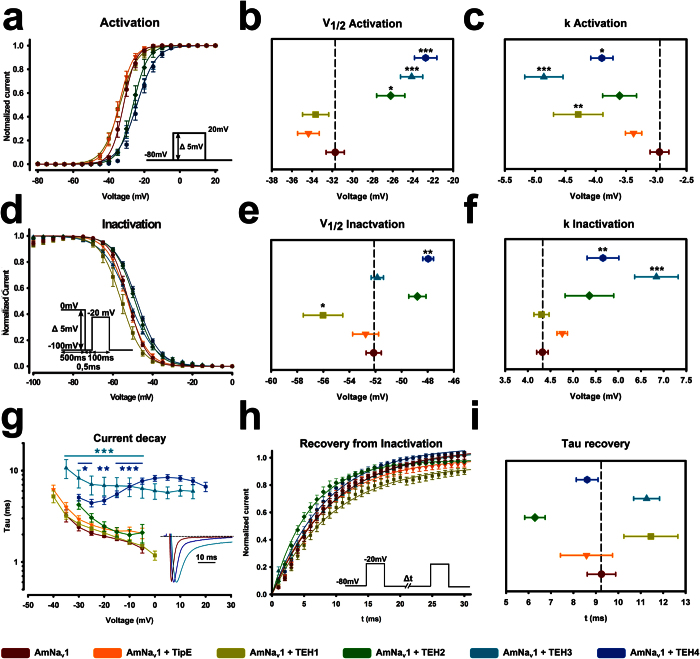
Effect of the different regulatory subunits on the biophysical properties of AmNa_V_1. **(a)** The voltage-dependence of activation (G–V). Activation curves were derived from I–V curves (n = 7–14). The I–V curves were obtained using the protocol shown in the inset. The peak current measured at each potential was then divided by (V–V_rev_), where V is the test potential and V_rev_ is the reversal potential. The conductances were then normalized and fitted to a standard Boltzmann equation (see Materials and Methods). **(b–c)** Plots of the fitted equation activation parameters (V_1/2_ and k_v_). Statistical differences were tested using a one-way ANOVA with Bonferroni’s correction. **(d)** The voltage-dependence of steady-state inactivation. The amplitude of the peak current was plotted as a function of the voltage imposed during the conditioning pulse (n = 8–14) using the protocol in the inset. The data was then fitted to a Boltzmann equation (see Materials and Methods). **(e–f)** Plots of the inactivation parameters (V_1/2_ and k_v_) of the fitted equation. Statistical differences were tested using a one-way ANOVA with Bonferroni’s correction. **(g)** Time constants of current decay. The protocol in **(a)** was used to generate transient sodium currents at different voltages. The decay of the transient current was fitted with a single exponential. For each condition, the time constant extracted from the fit was plotted as a function of the voltage imposed (n = 7–14). Typical raw data obtained as a result of a depolarization to −20 mV is shown in the inset to illustrate the different decay kinetics. **(h)** Recovery from inactivation. The amplitude of the peak current measured during the second pulse was divided by the amplitude measured during the first pulse and was then plotted against the time interval between the pulses (n = 8–14) using the protocol in the inset. **(i)** The resulting curves were fitted with a single exponential in order to compare the time constants. A statistical analysis using one-way ANOVA with Bonferroni’s correction was performed to assess significant differences between the oocytes expressing regulatory subunits and oocytes expressing AmNa_V_1 alone. *p < 0.05, and ** and ***p < 0.01 and 0.001.

An analysis of the activation properties of the AmNa_V_1 channel in the presence of the various regulatory subunits showed that the TEH2, TEH3, and TEH4 subunits shift the voltage-dependent activation of the channel to the right (Fig. 4a). When fitted to a Boltzmann function, this shift in the voltage-dependence of activation is represented by a shift of V_1/2_ toward more positive values (Fig. 4b). The TEH1, TEH3, and TEH4 subunits also had a significant effect on the slope factor of the Boltzmann curve (Fig. 4c), causing a less steep voltage-dependence of activation curve than the curve for the AmNa_V_1 channel alone. Overall, our data indicates that activation of the channel is slightly hindered in the presence of TEH2, TEH3, and TEH4, and is slightly facilitated in the presence of TEH1.

#### Voltage dependence of fast inactivation of the AmNa_V_1 channel

The voltage-dependence of fast inactivation of the AmNa_V_1 channel was also fitted with a Boltzmann function. The voltage of half-maximal inactivation (V_1/2_) in the absence of subunits was −52.1 ± 0.6 mV and the slope factor (k) was 4.3 ± 0.1 mV (n = 11).

The voltage-dependence of fast inactivation of the channel in the presence and absence of the regulatory subunits was evaluated using a standard two-pulse protocol. The current measured during the test pulse was plotted as a function of the voltage imposed during the conditioning pulse (Fig. 4d). Like the voltage-dependence of activation, the voltage-dependence of inactivation was fitted to a two-state Boltzmann function. A statistical analysis revealed a negative shift of the half-maximal voltage of inactivation (V_1/2_) in the presence of TEH1 and a positive shift in the presence of TEH4 (Fig. 4e). In addition, the slope factor (k) was higher in the presence of TEH3 and TEH4 (Fig. 4f). Overall, this means that TEH1 increases the steady-state inactivation of the channel while TEH4 decreases the steady-state inactivation. Differences in the time course of current decay were also observed (Fig. 4g). Fast inactivation was the main determinant of current decay. As illustrated by the raw data presented in Fig. 3a, the fast inactivation kinetics of the AmNa_V_1 channel were much slower in the presence of the TEH3 and TEH4 regulatory subunits, but were not affected by TipE, TEH1, or TEH2.

#### Recovery from inactivation

Recovery from inactivation was also evaluated using a two-pulse protocol. Briefly, the relative amplitude of the test pulse was plotted as a function of the Δt separating the conditioning and test pulses. The data points were fitted with a double exponential function. In the absence of the regulatory subunits, the faster exponential had a relative weight of 0.85 ± 0.05 and time constant (τ) of 8.2 ms ± 0.5 ms (n = 11). The slower exponential had a relative weight of 0.15 ± 0.05 and time constant (τ) of 80 ms ± 30 ms (n = 11). Despite slight apparent differences in the recovery from inactivation curves (Fig. 4h), no statistically significant differences were observed in the presence of the regulatory subunits (Fig. 4i).

#### Honeybee Na_V_1 single channel conductance

The single channel conductance of the honeybee Na_V_1 channel co-expressed with the TipE regulatory subunit was investigated using the patch clamp technique in the cell-attached configuration. Raw current traces were obtained at different voltages (Figure S4a). All-points histograms were then created in order to extract the mean single channel current amplitudes (Figure S4b). The extracted values were plotted as a function of the voltage imposed (Figure S4c). The data was fitted to a linear equation, yielding a slope value of 24.5 ± 0.2 pS for single channel conductance.

### Pharmacological characterization

#### TTX sensitivity

Mammalian sodium channels are often divided into two groups that seem to have branched early in evolution: TTX-sensitive and TTX-insensitive channels^42^. The pharmacological characterization of uncharted sodium channels often begins with a determination of their TTX sensitivity. We evaluated the TTX sensitivity of the AmNa_V_1 channel and its TipE regulatory subunit. As expected, based on the high level of conservation of the DEKA and EEDD clusters, TTX inhibited sodium currents in a concentration-dependent manner (Figure S5a). Current inhibition expressed as the fraction of channels blocked as a function of TTX concentration was then plotted and fitted with the Hill equation (Figure S5b). This procedure resulted in a good-quality fit (R^2^ = 0.9992), with an evaluated half maximal inhibitory concentration of approximately 0.7 nM (IC_50_ = 0.7 nM), which is similar to that of the *Drosophila* Na_V_1 channel^23^, indicating that the honeybee channel was TTX sensitive. This was also predicted by the presence of an aromatic amino acid at position 449 (Y449). Other TTX-sensitive sodium channels usually feature an aromatic residue in the corresponding position. Substitution of the corresponding residue for an aromatic amino acid in TTX-resistant channels increases the sensitivity of the channels to TTX^43^,^44^.

#### Sensitivity to pyrethroids

To determine whether the honeybee Na_V_1 channel could be used to test the sensitivity of the honeybee for pyrethroid insecticides, we tested the effect of permethrin and fenvalerate, respectively type I and type II pyrethroid insecticides.

**Figure 5:**
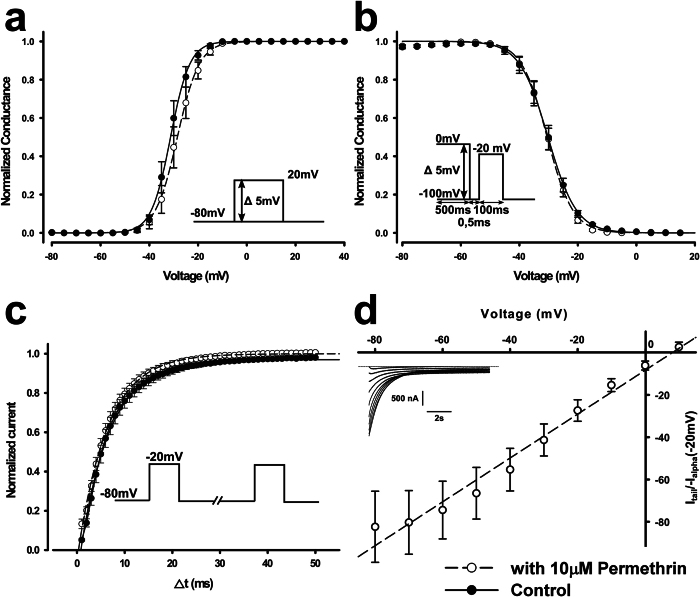
Effect of permethrin on the honeybee Na_V_1 channel. (**a**–**c**) The presence of 10 μM permethrin had no significant effect on the voltage-dependence of activation (**a**), the voltage-dependence of steady-state inactivation (**b**), or the recovery from inactivation (**c**) of AmNa_V_1 channels co-expressed with TipE. It did, however, induce a tail current. The maximal amplitude of the tail current after 200 conditioning pulses was recorded at different voltages (**d**). The normalized amplitude was plotted as a function of the voltage imposed during the recording (n = 9). The data was then fitted with a linear function (R^2^ = 0.98, y0 = −8.4%, slope = 1.0%/V).

Adding 10 μM permethrin to the extracellular medium did not induce statistically significant shifts in activation, inactivation, or recovery from inactivation (Fig. 5a–c). Following conditioning pulses, extracellular permethrin induced a tail current that exhibited a linear voltage-dependence and an inversion at approximately +10 mV (Fig. 5d). A similar reversal potential was also observed for the transient current elicited in response to the pulse protocol used to create the G–V curve shown in Fig. 4a, indicating that the transient current and the tail current have similar selectivity and are most likely of the same nature.

**Figure 6:**
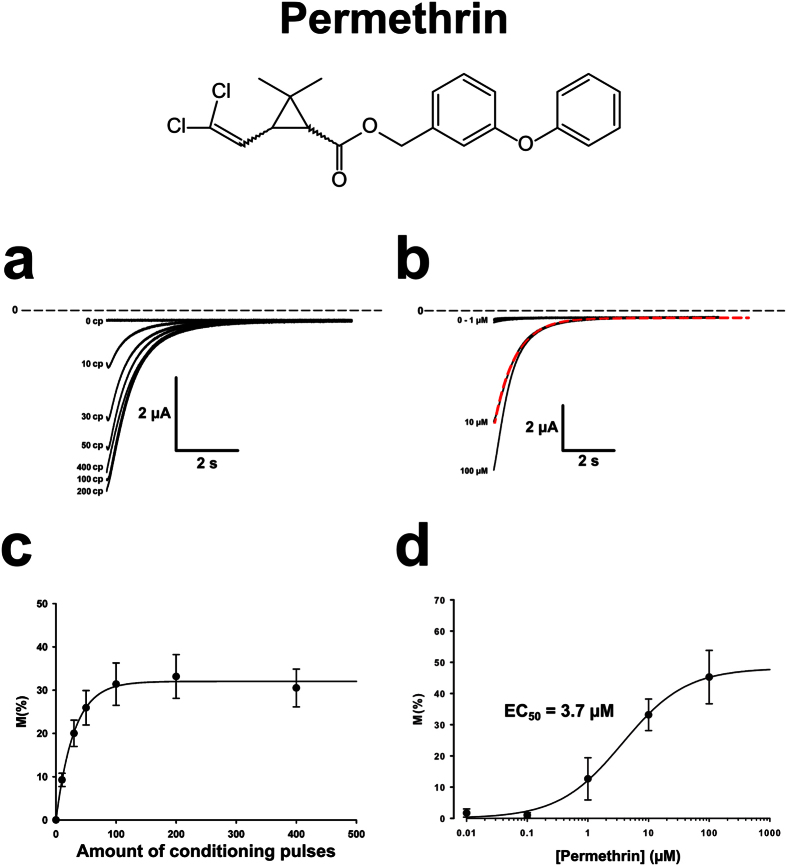
Permethrin induced tail currents in oocytes expressing AmNa_V_1. The presence of 10 μM permethrin induced tail currents in oocytes co-expressing the AmNa_V_1 channel and the TipE regulatory subunit. (**a**) The amplitude of the tail current was highly dependent on the number of depolarizations imposed before the recording (pulses from −80 mV to −20 mV repeated at a frequency of 100 Hz). (**b**) The amplitude of the tail current was also dependent on the concentration of permethrin in the extracellular medium. The red dotted line represents the fit of the tail current at 10 μM. (**c**) The number of conditioning pulses (CP) required to attain the maximal amplitude of the tail current in the presence of 10 μM permethrin was then determined (n = 4). (**d**) The fraction of channels modified by permethrin (M) was calculated using equation 1 in the supplemental methods and plotted as a function of the dose applied using 200 conditioning pulses. The data was then fitted with a Hill curve in order to extract the Hill coefficient (h = 0.83) and the half maximal effective concentration (EC_50_ = 3.7 μM). The error bars represent the SEM.

Permethrin induced tail currents following activation, indicating that it traps the channel in its open state. The amplitude of the tail current increased as a function of the number of conditioning pulses (Fig. 6a) and the concentration of permethrin (Fig. 6b), indicating that this insecticide binds to the channel in its open state. In order to characterize the affinity of the channel for permethrin, we measured the amplitude of the tail current using varying numbers of conditioning pulses. Plotting the amplitude of the tail current as a function of the number of conditioning pulses showed that the maximal effect is reached after 200 pulses (Fig. 6c). Determining the number of pulses needed to reach the maximal effect made it possible to plot a dose-response curve (Fig. 6d). When fitted with the Hill equation, the curve yielded an R^2^ of 0.998, a Hill coefficient of 0.83, and an EC_50_ of 3.7 μM.

**Figure 7:**
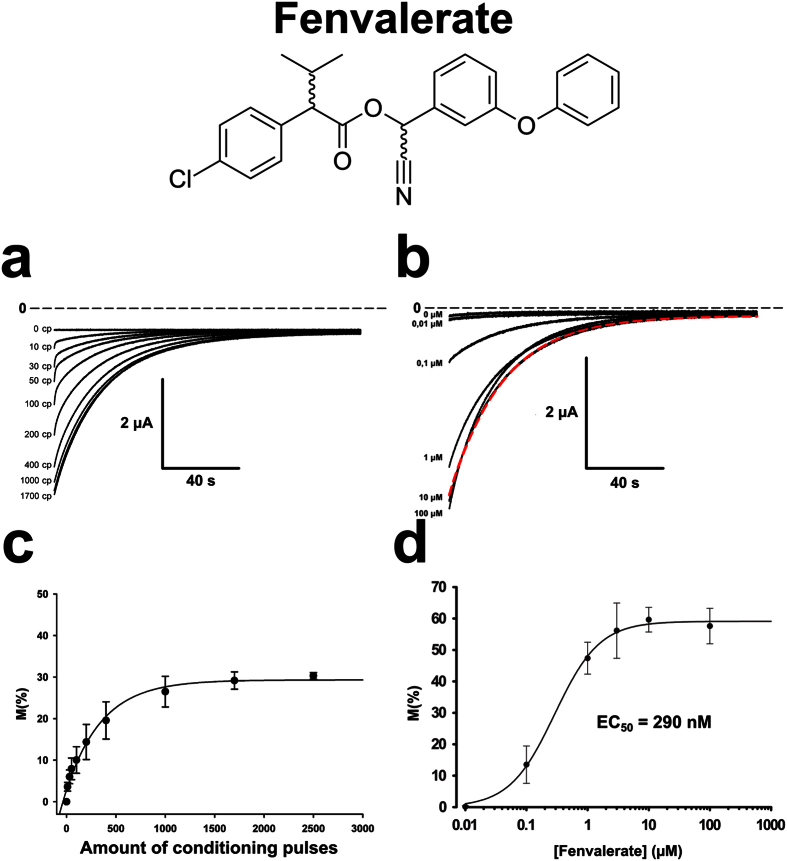
Fenvalerate induced tail currents in oocytes expressing AmNa_V_1. The presence of 10 μM fenvalerate induced a tail current in oocytes co-expressing AmNa_V_1 and its TipE regulatory subunit. (**a**) The amplitude of the tail current was highly dependent on the number of depolarizations imposed before the recording (pulses from −80 mV to −20 mV repeated at a frequency of 100 Hz). (**b**) The amplitude of the tail current was also dependent on the concentration of fenvalerate in the extracellular medium. The red dotted line represents the fit of the tail current at 10 μM. (**c**) The number of conditioning pulses (CP) required to attain the maximal amplitude of the tail current in the presence of 10 μM fenvalerate was then determined (n = 3). (**d**) The fraction of channels modified by fenvalerate (M) was calculated using equation 1 in the supplemental methods and plotted as a function of the dose applied using 1700 conditioning pulses. The data was then fitted with a Hill curve in order to extract the Hill coefficient (h = 1.18) and the half maximal effective concentration (EC_50_ = 290 nM). The error bars represent the SEM.

Fenvalerate induced similar tail currents that depended on the number of conditioning pulses (Fig. 7a) and the concentration of this insecticide (Fig. 7b). Determining the number of conditioning pulses needed for maximal effect (Fig. 7c) was necessary in order to create a dose-response curve for fenvalerate (Fig. 7d). Fitting the data to the Hill curve yielded an R^2^ of 0.9987, a Hill coefficient of 1.18, and an EC_50_ of 290 nM.

Comparing the results obtained from the application of permethrin and fenvalerate showed that more conditioning pulses were required to reach a maximal effect with fenvalerate. However, fenvalerate induced larger tail currents and had a higher affinity for the AmNa_V_1 channel than permethrin. In addition, the tail currents induced by fenvalerate took longer to deactivate (Figs 6b,7b). The deactivation of permethrin-induced tail currents was fitted to a single exponential with a time constant of 0.93 s ± 0.11 s. The deactivation of the fenvalerate-induced tail currents was better fitted to a double exponential. The relative weight of the slow component was 85.4% ± 3.6%. The time constants for both exponentials were 9.9 s ± 2.4 s and 31.8 s ± 1.8 s respectively.

### Structural model and molecular docking

A structural model of the AmNa_V_1 channel was generated using Modeller^45^. The open-activated equilibrated structure of the Na_V_Ab bacterial channel was used as a template^46^. This structure was preferred to the recently published partially activated crystal structure^47^ since our results indicated that pyrethroid insecticides bind to the activated/open channel. The homology model was then equilibrated for 20 ns using molecular dynamics simulations of the channel embedded in a solvated and ionized POPC model membrane.

The resulting structure was used for docking experiments to determine whether our model could be used for *in silico* compound screening. Since a binding site for pyrethroid insecticides was recently identified in the homologous *Aedes aegypti* Na_V_1 channel^48^, and since the amino acid sequence of the proposed binding site in the AmNa_V_1 is the same as that that of the *A. aegypti* channel, a suitable model for *in silico* compound screening should recapitulate this fact. The sensitivity of the channel to pyrethroid insecticides reported here also provided support for the existence of such a site.

**Figure 8:**
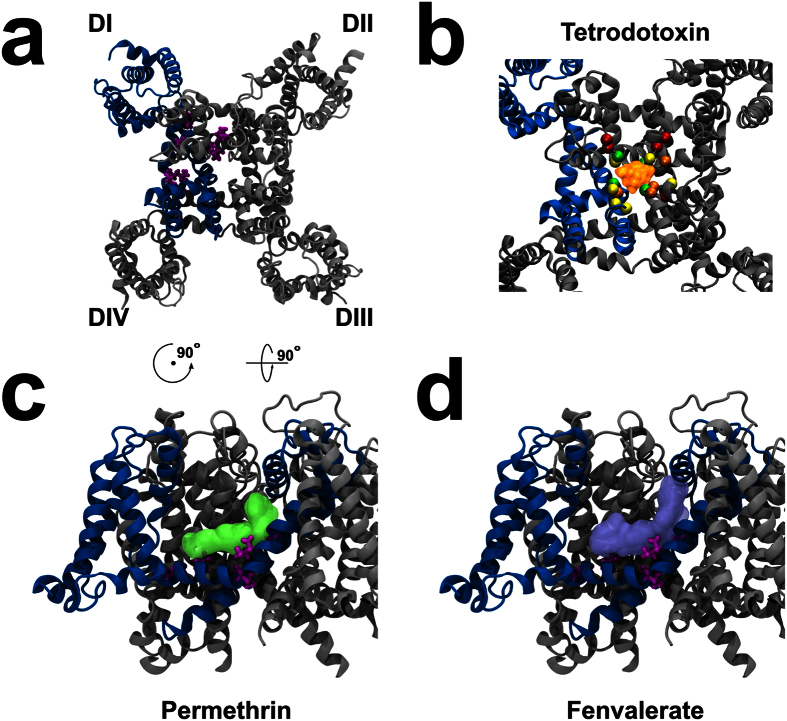
Structural model and docking poses AmNa_V_1. (**a**) Top view of one of the poses used for the docking calculations. The protein is in grey with the exception of the first domain, which is in blue. The residues involved in the binding site mapped by Du *et al*. in the *A. aegypti* channel are in purple. (**b**) A close-up view of the pore from the top, with the most populated docking pose for TTX. The most populated docking pose for TTX is in orange. The α-carbon of the residues involved in the DEKA motif of the selectivity filter are in green (D386, E989, K1496 and A1789). The residues located within 4.5 Å of the toxin which are known to contribute to TTX binding in mammalian sodium channels are displayed in yellow (Y387, W388, E389, G988, G1497 and D1792). The α-carbon of other residues known to contribute to TTX binding in mammalian sodium channels according to the model published by Tikhonov and Zhorov but not located within 4.5 Å of the TTX cluster are displayed in red (W990, E992, W1498, Q1500 and W1791). The α-carbon of the residues located within 4.5 Å of the TTX cluster but not considered to play a major role in TTX binding according to Tikhonov and Zhorov are shown in orange (D386, Y387, E389 E989, K1496, A1789 and D1792). (**c**) The most populated docking poses for permethrin are shown on a side view of the protein. (**d**) The most populated docking poses for fenvalerate are shown in the same view. The residues in purple in c and d are the residues involved in the binding site mapped by Du *et al*.

Directed docking experiments using a large box (40 Å × 20 Å × 30 Å) centered on the proposed binding site revealed binding sites for permethrin and fenvalerate. Clustering all the poses obtained for permethrin (7799) and fenvalerate (7816) revealed that, in over 70% of the poses, the ligands would be in contact with residues that would induce knock-down resistance to pyrethroids if mutated (Fig. 8). This indicated that the model presented here can be used as an *in silico* tool to determine whether pyrethroid derivatives induce undesirable effects in honeybees.

A directed docking experiment using a larger box (40 Å × 40 Å × 70 Å) centered on the pore of the channel was also performed to determine whether the model could account for the high TTX-sensitivity of the channel. We assessed the potential of residues of being implicated in the binding of TTX based on their proximity with the principal toxin conformation cluster, using a cutoff of 4.5 Å to discriminate between binding and non-binding residues. The model indicated that the residues of main selectivity filter (D386, E989, K1496 and A1789) are in close proximity of the most represented TTX conformation cluster (Fig. 8b). This was expected given that the binding site identified in mammalian sodium channels is also located in this vicinity^38^. Additionally, two of the four residues which constitute the secondary selectivity filter were close to the ligand, namely E389 and D1792. Again, the corresponding residues in mammalian sodium channels were shown to contribute to TTX binding by Terlau *et al*.. We also show that the Y387 seems to be implicated in TTX binding. This residue aligns with C373 in hNa_V_1.5. Interestingly, some mutations on the C373 residue are known to increases the sensitivity of the human channel to TTX^43^,^44^. Furthermore, the residues W388, G988 and G1497 were also in close proximity to TTX. The corresponding residues have been shown to contribute to TTX binding in other investigations on mammalian channels^38^,^49^. As could be expected, some differences existed between our docking experiments on insect channel compared to previous docking experiments on mammalian channels. Indeed, according to a model published by Tikhonov and Zhorov, the residues corresponding to the residues W990, E992, W1498, Q1500 and W1791 would contribute to TTX binding^49^. In our model, those residues were not within 4.5 Å of the ligand. Residues within 4.5 Å of the TTX cluster would rather include Q385, W402, Q1491, S1788, G1790 as well as the residues reported above (D386, Y387, E389 E989, K1496, A1789 and D1792). Although both models rely on docking experiments unconfirmed by mutagenesis, this could indicate that the TTX binding site could be slightly closer to the primary selectivity filter in AmNa_V_1, when compared to mammalian Na_V_1.

## Discussion

The sequence of the *Apis mellifera* sodium channel (AmNa_V_1) features four homologous domains, a selectivity filter, a hydrophobic cluster in the DII/DIII linker, and a putative phosphorylation site. Similar motifs are present in mammalian and other insect sodium channels. The sequence variations identified here are all located in the intracellular loops, which often feature protein-protein interaction or phosphorylation sites. Further investigations are warranted to determine the functional impacts and physiological implications of these splice variants.

The honeybee Na_V_1 channel shares striking similarities with other cloned insect sodium channels. A comparison with the Na_V_1 sequences of other pyrethroid-sensitive insects showed that the sequence reported here shares 78%, 80%, and 82% identity with the amino acid sequences from *Musca* domestica (house fly), *Drosophila* melanogaster (fruit fly), and A. *aegypti* (yellow fever mosquito) (accession numbers NP_001188635.1, AAB47605.1, and ACB37024.1, respectively). Pyrethroids are used to control these three insects and the diseases they spread^10^.

Interestingly, the honeybee Na_V_1 channel only shares 58% identity with the *Varroa destructor* Na_V_1 channel (accession number AAP13992.1). This mite is a major vector of diseases affecting bees. Further investigations using both channels are required to determine whether there are any differences in the binding sites and affinities of these channels for pyrethroids. The results of these investigations could lead to the development of highly specific insecticides for in-hive use in order to control infections propagated by *V. destructor*.

Like the regulatory subunits of the *D. melanogaster* Na_V_1 channel^24^, the TipE and TEH subunits increased the expression of the honeybee Na_V_1 channel (Fig. 3).

The honeybee genome, like the *D. melanogaster* genome, codes for five regulatory subunits. While they share relatively low identity, the regulatory subunits of Na_V_1 channels appear to share a common ancestor with mammalian K_Ca_β regulatory subunits^24^. It would thus be interesting to determine whether the TipE and TEH subunits interact with honeybee K_Ca_α channels.

RT-PCR experiments showed that the RNA corresponding to all the proteins investigated are expressed almost ubiquitously in honeybee tissues, indicating that the AmNa_V_1 channel may be involved in a wide variety of physiological functions in the honeybee. Curiously, earlier reports indicate that no sodium current were detectable in the honeybee’s muscle fibres^50^. Caution is warranted in the interpretation of RT-PCR results as we did not attempt to determine if the detection of RNA in those tissues is accompanied by the expression of proteins. Further experiments are warranted to determine if AmNa_V_1 proteins are expressed in all those tissues and whether they possess a significant physiological role.

We observed very few sequence variations for Na_V_1, TipE, and TEH4. However, the sequence variations we mapped were seen in total RNA preparations from honeybee heads. Further investigations should focus on mapping all the sequence variations for the proteins reported here using other total RNA preparations. Examining the expression of sequence variants in specific tissues would be the first step toward determining their physiological roles.

The biophysical characterization of the *Apis mellifera* Na_V_1 channel with and without its regulatory subunits provided a brief overview of the basic properties of the channel. The regulatory subunits can be divided into two subfamilies based on their effect on the Na_V_1 channel. TipE, TEH1, and TEH2 did not change or increase steady-state inactivation while TEH3 and TEH4 hindered entry into the inactivated state. TEH3 and TEH4 also hindered activation of the channel and had a marked effect on current decay. The proposed division into two subfamilies is supported by the sequence analyses we performed. The phylogenetic tree indicated that the sequences of TEH3 and TEH4 are more similar to each another than to the other regulatory subunits. They are also the only proteins of the family to feature EGF-like domains.

The experiments reported here provide all the protocols and results required to test the toxicity of compounds for honeybees. Similar experiments could also be performed on other cloned insect channels and could be compared to the results from the honeybee channel to make sure that new insecticides are toxic for other insects, but not the honeybee.

The AmNa_V_1 channel was very sensitive to TTX. Similar affinities have been reported for other Na_V_1 channels^23^ and TTX-sensitive mammalian sodium channels (Na_V_1.1–1.4 and Na_V_1.6–1.7)^51^. The TTX sensitivity of the honeybee Na_V_1 channel was to be expected as most of the amino acid known to determine TTX sensitivity in mammalian channels are conserved in the AmNa_V_1 channel. Indeed, the secondary selectivity filter is mostly conserved, the primary DEKA filter is conserved and there is an aromatic residue at the position following the D of this filter (Y387). Moreover, other residues located between the filters which are known to contribute to TTX binding are also conserved. However, our docking experiments has shown that residues located outward of the secondary selectivity filter may not contribute to TTX binding in AmNa_V_1. Other residues located closer to the primary selectivity filter could be implicated in TTX binding. This displacement of the TTX binding site slightly closer to the DEKA cluster compared to TTX sensitive mammalian Na_V_1 channel could be a feature of insect Na_V_1 channels. Further mutagenesis experiments are required to confirm this hypothesis.

Permethrin and fenvalerate induced tail currents following activation. However, the EC_50_ for permethrin was shifted by more than an order of magnitude, indicating that type II pyrethroids may have higher affinities for the honeybee Na_V_1 channel. While we calculated an affinity of approximately 3.7 μM for AmNa_V_1, this does not mean that permethrin is not chronically or acutely toxic for the honeybee. Rather, it indicates that careful dosage may help minimize the impact on honeybees, although chronic exposure may still be deleterious^31^. The EC_50_ we measured is very close to the EC_50_ for permethrin on the sodium current of honeybee antennal olfactory receptor neurons (3.5 μM)^30^, indicating that the undesirable effect of permethrin on the olfactory functions of the honeybee may be exclusively due to the binding of the compound to the Na_V_1 channel. It also indicates that the *in vitro* technique proposed here replicates the results obtained from more complicated setups.

Fenvalerate had an EC_50_ of approximately 290 nM for AmNa_V_1. This higher affinity at lower concentrations than permethrin may make fenvalerate less suitable for agricultural uses. Fenvalerate also induced tail currents that decay less rapidly than the currents induced by permethrin, indicating that it could potentially be more toxic for honeybees. However, the LD_50_ of *Apis mellifera* reported for fenvalerate is twice that of permethrin^52^. The reason for this discrepancy might for instance be found either in detoxication capacities, in the existence of a more toxic metabolite or in the existence of a secondary target for permethrin. Therefore, although our model may be used as a precise and relatively fast way to assess the toxicity of chemical compounds for honeybees, this method would be complementary to *in-vivo* testing.

Interestingly, using similar protocols, other insect sodium channels were found to have similar susceptibility for pyrethroids^53^. However, the *Drosophila* Na_V_1 channel seems more affected by permethrin than the honeybee channel (EC_50_ = 12 nM for the *Drosophila* channel^54^ vs. 3.7 μM for the honeybee channel). This difference may be due in part to differences in the experimental procedures. Indeed, Vais *et al*. used ATX-II to inhibit inactivation while this toxin was not used in our study. In investigations where ATX-II was not used, the *Drosophila* channel displayed half maximal effective concentration superior to 0.01 μM^55^. Similarly, the EC_50_ reported for deltamethrin on the *Drosophila*’s channel in the presence of ATX-II is around 4.7 nM whereas no response was recorded at 100 nM in the absence of ATX-II^56^. Some key residues in the sequence of the honeybee Na_V_1 channel could also explain potential differences between the affinities of the honeybee and *Drosophila* channels. Rinkevich *et al*. summarized the state of knowledge about 38 mutations associated with knock-down resistance to pyrethroids in arthropods^21^. Five amino acids in the sequence of the honeybee Na_V_1 channel are suggestive of mutations associated with knock-down resistance to pyrethroids in arthropods. While these five amino acids are unlikely to account for the shift in sensitivity of the honeybee channel compared to the *Drosophila* channel, investigations focused on these residues are warranted. The E485K mutation (using *M. domestica* numbering) has been associated with knock-down resistance in *Blattella germanica* in the background of L1014F and L1014F+C785R^57^. The corresponding residue in the honeybee sequence is also negatively charged (D489). It is thus unlikely that this residue contributes significantly to a change in affinity for pyrethroids.

The L1596P mutation (using *M. domestica* numbering) in *V*. destructor induces a lower affinity for pyrethroids^58^. The corresponding residue in the honeybee is P1595. Interestingly, this residue is also a proline in other known insect Na_V_1 channels. This may explain potential differences in the affinity of the *V. destructor* Na_V_1 and the honeybee’s Na_V_1 for pyrethroids, but not between the honeybee and *Drosophila* channels since the proline residue is present in both these species.

The A1101T, A1215D, and M1823I mutations (using *M*. domestica numbering) have also been associated with knock-down resistance phenotypes. However, one should be careful in drawing conclusions from these results since none of these mutations have been confirmed *in vitro*. Further information on these mutations and the corresponding residues in the AmNa_V_1 channel sequence can be found in the Supplemental Information section (Supplemental Table 1).

We also report here the creation of a structural model of the honeybee Na_V_1 sodium channel, which paves the way to *in silico* screening of compounds that can bind to the pore or to the pyrethroid binding site. This structural model may be used as a first step in the intelligent design of compounds intended either to target or to avoid targeting the honeybee Na_V_1 sodium channel. Our model indicated that the binding site for pyrethroids in the honeybee sodium channel is similar to the site recently identified in *A. aegypti*^48^. This may be an obstacle for the creation of insecticides that specifically target the Na_V_1 channels of insect pests but not the honeybee. However, given the low homology between the Na_V_1 channels of the honeybee and *V. destructor*, pyrethroid derivatives could be used to target this parasite which now invades the hives of both eastern and western honeybees^59^.

The binding poses showed pyrethroid insecticides in close proximity to the residues corresponding to those mapped by Du *et al*.^48^. The distance of docked compounds from these residues could thus be used as an objective criterion to predict potential interactions with the Na_V_1 channel.

We report the creation of new *in vitro* and *in silico* tools to test the toxicity of compounds for honeybees. These tools could be used to screen insecticides for agricultural and in-hive use. Compounds that induce statistically significant changes to the properties of the AmNa_V_1 channel at their recommended doses must be used with care. Efforts to reduce or eliminate toxic products from the environment should help alleviate pressure on honeybee colonies. Regardless of whether the cause of CCD is an infection, the overuse of chemicals, or a combination of these causes, taking the toxicity of certain compounds for honeybees into account should help in the fight against the disappearance of our principal pollinator.

## Material and Methods

### Solutions and reagents

The Ringer’s bath solution was composed of 116 mM NaCl, 2 mM KCl, 2 mM CaCl_2_, 2.9 mM MgCl_2_, and 5 mM HEPES. The pH was adjusted to 7.6 at 22 °C using 1 M NaOH.

The hyperosmotic solution used for single-channel recordings was composed of 200 mM K-aspartate, 20 mM KCl, 1 MgC1_2_, 10 mM EGTA, and 10 mM HEPES (pH 7.5). For cell-attached experiments, the bath solution was composed of 100 mM K-aspartate, 50 mM KCl, 1.5 mM CaCl_2_, 1 mM MgCl_2_, 10 mM glucose, and 10 mM HEPES (pH 7.4). The pipette solution was composed of 150 mM NaCl, 2 mM KCl, 1.5 mM CaCl_2_, 1 mM MgCl_2_, 10 mM glucose, and 10 mM HEPES (pH 7.4).

The OR_3_ solution was composed of a 1:2 dilution of Leibovitz’s L-15 medium supplemented with 15 mM HEPES and 50 mg/ml of gentamycin. The pH was adjusted to 7.6 at 22 °C using 1 M NaOH^60^.

The OR_2_ solution was composed of 82.5 mM NaCl, 2.5 mM KCl, 1 mM MgCl_2_, and 5 mM HEPES. The pH was adjusted to 7.6 at 22 °C using 1 M NaOH.

All the chemicals and drugs were from Sigma, except for tetrodotoxin, which was from Latoxan (France) and Leibovitz’s L-15 medium, which was purchased from Invitrogen (USA) in powder form. All the experiments were carried out at 22 °C.

### Sequencing and cloning

The regulatory subunits were cloned in the pPol_Not1 vector. Two overlapping fragments of the AmNa_V_1 channel were cloned in the TOPO vector. Details about the generation of mRNA for oocyte injection and about the molecular cloning are available in the supplemental information section.

### RT-PCR

Total RNA from honeybee tissues was extracted using Trizol kits (Sigma). cDNA preparations were then produced using Transcriptor first strand cDNA synthesis kits (Roche). cDNA preparations corresponding to the Na_V_1, TipE, and TEH1-4 proteins were obtained by PCR amplification using oligonucleotide primers. The resulting cDNA preparations were run on agarose gels to determine whether the genes of interest were expressed in the tissues under investigation. Controls were performed with total RNA preparations using the same protocol but without reverse transcriptase. Primer sequences are available in the supplemental information section.

### *Xenopus* oocytes

All experimental procedures involving *Xenopus* oocytes were approved by the Université Laval Institutional Animal Care Committee in line with the principles and guidelines of the Canadian Council on Animal Care (Approval 2011155-1). They were prepared as described previously^60^. Briefly, oocytes were surgically removed from frogs anesthetized with MS-222. They were then treated with 2 mg/ml collagenase (Collagenase type 1A from *Clostridium histolyticum*, Sigma) in OR_2_ solution for 1 h. They were then incubated for at least 1 h at 18 °C in OR_3_ solution. Stage IV and V oocytes were microinjected with mRNA corresponding to AmNa_V_1 and the selected regulatory subunit (1 μg/μl for AmNa_V_1 and 0.3 μg/μl for regulatory subunits, 50 nl/oocyte). The injected oocytes were incubated at 18 °C in OR_3_ solution for at least 12 h before taking recordings.

### Electrophysiology

Macroscopic currents from mRNA-injected oocytes were recorded in Ringer’s solution using the two-microelectrode voltage-clamp technique. The membrane potential for the two-microelectrode voltage-clamp technique was controlled using a Warner oocyte clamp (Warner Instrument Corp.). The currents were filtered at 5 kHz (−3 dB; four-pole Bessel filter). The headstage of the Warner amplifier was attached to a plastic pool containing a 3 M NaCl solution through a silver chloride wire connected to the bath solution using an agar bridge. The bridge contained 3% agar (w/v), 500 mM NMDG, and 10 mM HEPES (pH 7.4) and was threaded with a thin platinum/iridium wire to lower high-frequency impedance.

For the cell-attached patch experiments, the vitelline membrane was removed with forceps after briefly placing an oocyte in a hyperosmotic solution. Command pulses were generated and currents were recorded using pCLAMP software v10.3 and an Axopatch 200B amplifier (Molecular Devices). Patch electrodes (3–7 MΩ) fashioned from borosilicate glass (Corning 8161) were coated with HIPEC^®^ (Dow Corning) to reduce capacitance and noise emissions. Single-channel currents were filtered at 2 KHz and were sampled at 100 kHz. Traces were low-pass filtered at 1.5 kHz using a digital filter included in the Clampfit software. Capacity transients were eliminated by averaging recordings without openings and subtracting this average from all recordings.

### Data analysis and statistics

The electrophysiological data were analyzed using macros in Clampfit (pCLAMP v10.0; Molecular Devices) and custom scripts written using MATLAB (The MathWorks Inc.). The results are expressed as means ± SEM. Statistical comparisons were performed using a one-way ANOVA with Bonferroni’s post hoc test in SigmaPlot (Systat Software). Differences were deemed significant at *p* < 0.05. The *p* values and number of measurements are indicated in the text or figure legends. Further information about the equations used for fits or to calculate the fraction of channels modified by pyrethroid insecticides are available in the supplemental information section.

### Sequence alignments and homology modeling

The best template for building a model of the AmNa_V_1 channel transmembrane domains was identified by a PSI-BLAST search of the Protein Data Bank database. Since the Na_V_Ab high-resolution structure released in 2011^47^ bore the transmembrane domains with the highest max score, the recently published activated-open structure was used as a template for the homology model^46^.

The multiple sequence alignments were performed using the Clustal Omega web server^61^. Before building the model, the highly disordered intracellular loop regions between the four channel subunits were removed from the alignment because no structural template was available for them.

A standard MODELLER^45^ routine was used to build a comparative model of the AmNa_V_1 channel comprised of the pore domain and the four VSD. The homology model provided a low-resolution starting structure and, given the high structural similarity between the VSD of all the possible templates in the Protein Data Bank (K_V_1.2, K_V_1.2/2.1, Na_V_Ab, and Na_V_Rh), the choice of the reference structure was not critical. Molecular dynamics simulations (see the following section) were used to relax the model in its membrane/solution environment.

### Molecular dynamics simulations

The AmNa_V_1 channel model was then inserted in a fully hydrated POPC bilayer. The system was equilibrated under normal constant temperature and pressure conditions (298 °K, 1 atm) in a 116 mM NaCl solution. To ensure correct reorganization of the lipids and solution, the positions of the phosphates in the lipids and the positions of all of the atoms in the channel were constrained during the first nanosecond. The protein was constrained during the second step. The harmonic constraints on the protein were progressively removed over a period of 8 ns during the third step. Lastly, a 20-ns unrestrained MD simulation of the entire channel was conducted to relax the system.

The MD simulations were carried out using NAMD2.9^62^. Langevin dynamics were applied to keep the temperature at 300 °K. The equations of motion were integrated using a multiple time-step algorithm^63^. Short- and long-range forces were calculated every first and second time-step, respectively, with 2.0-fs time-steps. Chemical bonds between hydrogen and heavy atoms were constrained to their equilibrium values. Long-range electrostatic forces were taken into account using the particle mesh Ewald approach^64^. The water molecules were described using the TIP3P model^65^. The simulation used the CHARMM27-CMAP force field with torsional cross-terms for the protein^66^ and CHARMM36 for the phospholipids^67^. A united-atom representation was adopted for the acyl chains of the POPC lipid molecules^68^. The simulations were performed on Temple University’s Owl’s Nest supercomputer.

### Docking calculations

All the known enantiomers for each ligand were docked separately to the equilibrated model. Given that the conformational flexibility of the channel influences ligand binding, the compounds were docked against a family of ten equilibrium conformations of the channel structures. The conformations were sampled in the last 2 ns of the corresponding equilibrium MD simulations.

Ten independent docking calculations were performed for each ligand using AutoDock Vina 1.1.2^69^. Each docking calculation yielded approximately 20 different docking poses. The ligand was docked on the receptor structure by considering a 40 × 20 × 30 Å^3^ grid for the proposed binding site of pyrethroid insecticides and a 40 × 40 × 70 Å^3^ grid for the binding site in the pore domain. The grid spacing used was 0.45 Å for both binding sites. A total of 7799, 7816, and 7996 docking solutions were recovered for permethrin, fenvalerate, and TTX, respectively.

### Clustering

A cluster analysis was used to identify the binding sites of the ligands. Clustering was performed using the GROMOS algorithm in g_cluster with a geometric distance cutoff of 0.5 Å. This algorithm is described in Daura *et al*.^70^ Clusters were ranked by population. The top cluster for each ligand was considered as being in the putative binding site. For permethrin and fenvalerate, the top 3 and top 5 clusters, respectively, contained over 70% of the docking solutions. These clusters were considered as being in the binding site since they were in contact with residues corresponding to the binding site identified by Du *et al*.^48^ (Supplemental Table 2) and displayed some overlap with the most populated cluster. The residues with at least one atom located within 4.5 Å of the ligand were considered as interacting candidates.

## Additional Information

**How to cite this article**: Gosselin-Badaroudine, P. *et al*. Characterization of the honeybee AmNa_V_1 channel and tools to assess the toxicity of insecticides. *Sci. Rep*. **5**, 12475; doi: 10.1038/srep12475 (2015).

## Supplementary Material

Supplementary Information
