# Supplementary material for: Characterization of the honeybee AmNaV1 channel and tools to assess the toxicity of insecticides
Source: Sci Rep. 2015 Jul 23;5:12475. doi: 10.1038/srep12475 (PMC4894402; doi:10.1038/srep12475)
Supplement: Supplementary Information [file srep12475-s1.pdf]

Supplemental Information:

Characterization of the honeybee $AmNa_v1$ channel and tools to assess the toxicity of insecticides

Pascal Gosselin-Badaroudine[1], Adrien Moreau[1], Lucie Delemotte[2], Thierry Cens[4], Claude Collet[5], Mathieu Rousset[4], Pierre Charnet[4], Michael L. Klein[2], and Mohamed Chahine[1,3]

[1]Centre de recherche, Institut universitaire en santé mentale de Québec, Quebec City, QC, Canada G1J 2G3

[2]Institute of Computational Molecular Science, Temple University, Philadelphia, PA 19122 USA

[3]Department of Medicine, Université Laval, Quebec City, QC, Canada G1K 7P4

[4]Centre de Recherche de Biochimie Macromoléculaire (CRBM), CNRS UMR 5237, 1919 Montpellier, France.

[5]INRA UR 406, Abeilles et Environnement, 228 Route de l'Aérodrome, Domaine Saint Paul - Site Agroparc, CS40509, 84914 Avignon Cedex 9, France

Short title:

Functional expression and characterization of the honeybee Na_v1 channel

Key words: Apis mellifera, voltage gated ion channel, voltage gated sodium channel, insecticide toxicity, Nav1

Correspondence to:
Mohamed Chahine, Ph.D.
Centre de recherche
Institut universitaire en santé mentale de Québec
2601 chemin de la Canardière
Quebec City, QC, Canada G1J 2G3
Telephone: 1-418-663-5747, ext.4723
Fax: 1-418-663-8756
Email: mohamed.chahine@phc.ulaval.ca

Supplemental results: sequence and features of the honeybee's Na_V1 channel regulatory subunits

Five regulatory subunits for the Na_V1 channel have been identified previously in *Drosophila Melanogaster* [1]. Interestingly, these subunits were reported to be similar to the regulatory subunits of mammalian calcium-activated potassium channels (K_{Ca}) which are distant cousins of Na_v channels in the VGL-chanome [2]. Screening a HMM protein profile inferred from $K_{Ca}\beta$ subunits against the bee proteome resulted in the successful identification of the TipE protein and its homologs (e-values from 3,5E-66 to 5,8E-68). Unlike the Na_V1 sequence characterization, the TipE and TEH sequence characterizations were performed with the jackhammer algorithm. This algorithm consists in iterative hmmsearch-like queries. Each new query uses a protein profile inferred from the selected proteins associated with the significant matches of the previous query. This procedure was performed in order to make sure that all TipE homolog proteins were identified from the bee proteome. Using a similar approach with a protein profile inferred from mammalian regulatory subunits of Na_v channels did not highlight any homologous protein in the bee proteome.

Comparison of the identified subunits with the known $K_{Ca}\beta$ (*KCNMB*) subunits shows that, these proteins belong to a single protein family **(Figure 1D)**. Moreover, based on sequence homology, the TEH family is divided into two subfamilies. TEH3 and TEH4 seem to feature sequence motifs slightly different from TipE, TEH1 and TEH2

All the regulatory subunits of $AmNa_V1$ are located within a span of 2 686 kB in the LG1 chromosome **(Figure S2C)**. The TipE, TEH2, TEH3 and TEH4 genes are all located in closer proximity (within 74 kB). All the TEH subunits are oriented in the same direction. Similar arrangements have been reported for the TipE and TEH subunits of the *Drosophila Melanogaster*, *Drosophila Pseudoobscura*, *Drosophila Yakuba* and *Anopheles gambiae* [1]. Such an arrangement either points to early gene duplication and inversion events in the phylogeny or to common regulation of the genes.

Using the SOSUI web server [3], we identified two putative transmembrane segments in all regulatory subunit but TEH1. Given that all the putative transmembrane segments identified are in the same regions and that drosophila's TEH1 subunit is thought to feature

two transmembrane segments[1], it is probable that the honeybee's TEH1 also has two transmembrane segments in the same regions **(Figure S3)**.

As noted by Derst and collaborators for the drosophila's TipE and TEH subunits[1], some cysteines thought to be involved in the formation of disulphide bridges in $hK_{Ca}\beta2$ are conserved in the honeybee's Na_V1 regulatory subunits[1]. Other cysteines not found in $hK_{Ca}\beta2$ seem to be highly conserved in both drosophila's and honeybee's regulatory subunits **(Figure S3A-B)**. Those residues may be involved in the formation of disulphide bridges as well. Furthermore, TEH3 and TEH4 both feature two EGF-like domains. Those domains are typically associated with protein-protein interactions and calcium binding. TEH3's first EGF-like domain seems to be involved in calcium binding as it features the D/N-x-D/N-E/Q-x_1-D/N*-x_2-Y/F consensus sequence (where x_1 and x_2 are of variable length and * represents β-hydroxylated residue). TEH4's first EGF-like domain, however, only features some residues of the consensus sequence.

No alternative sequences were found for the honeybee's TEH1, TEH2 and TEH3 subunits. Nevertheless, alternative sequences for TipE and TEH4 were found as a result of genomic variations and RNA editing **(Figure S2B)**.

References:

1 Derst, C., Walther, C., Veh, R. W., Wicher, D. & Heinemann, S. H. Four novel sequences in Drosophila melanogaster homologous to the auxiliary Para sodium channel subunit TipE. *Biochem Biophys Res Commun* **339**, 939-948, doi:10.1016/j.bbrc.2005.11.096 (2006).

2 Yu, F. H. & Catterall, W. A. The VGL-chanome: a protein superfamily specialized for electrical signaling and ionic homeostasis. *Sci Signal* **2004**, re15, doi:10.1126/stke.2532004re15 (2004).

3 Hirokawa, T., Boon-Chieng, S. & Mitaku, S. SOSUI: classification and secondary structure prediction system for membrane proteins. *Bioinformatics* **14**, 378-379 (1998).

Supplemental methods: cloning $AmNa_V1$ and its regulatory subunits

Total RNA from honeybee heads was extracted using Trizol kits (Sigma). cDNA was produced using Transcriptor first strand cDNA synthesis kits (Roche). The cDNA corresponding to two overlapping fragments of the Na_V1, the TipE, and TEH1-4 proteins was obtained by PCR amplification. Prior to insertion in a plasmid with a standard

digestion and ligation method, restriction sites were introduced at the beginning and end of each fragment cloned using PCR amplification with the following primers:

TipE :

Forward primer with **BamH1** restriction site:

GAGA**GGATCC**GCCGCCACCATGGCGGAGGAAAAGGAGAAG

Reverse primer with **HindIII** restriction site:

GAGA**AAGCTT**GCTTCAGACTGCCGCCGT

TEH1

Forward primer with **BamH1** restriction site:

GAGA**GGATCC**GCCGCCACCATGCGCGGGAGCAGCTCTGA

Reverse primer with **EcoR1** restriction site:

GAGA**GAATTC**TTACCTGAGATCGTTGCCCCTG

TEH2

Forward primer with **BamH1** restriction site:

GAGA**GGATCC**GCCGCCACCATGGGCGCCCAGGAAGAG

Reverse primer with **HindIII** restriction site:

GAGA**AAGCTT**TTACTCTTCCTTCCTTGCGTACTTGT

TEH3

Forward primer with **BamH1** restriction site:

GAGA**GGATCC**GCCGCCACCATGCCTAAGCAAGTGCCAGTG

Reverse primer with **EcoR1** restriction site:

GAGA**GAATTC**TCAGAGCGCCATAGCGGAGT

TEH4

Forward primer with **EcoR1** restriction site:

GAGA**GAATTC**GCCGCCACCATGGGTCGAAAGCATAAACG

Reverse primer with **HindIII** restriction site:

GAGA**AAGCTT**CTGCTAAAGCGCCATGCT

Nav1 fragment #1 (from ATG to the 4131th nucleotide, starting with A from ATG)

Forward primer with **Kpn1** restriction site:

GAGA**GGTACC**GCCGCCACCATGTCCGAAGATTCTGACTC

Reverse primer:

GCCAGCGCCACAGAGCGACGCTACGAAGTTAATGAGTGAC

Nav1 fragment #2 (from 4092th nucleotide to the stop codon)

Forward primer:

GTCACTCATTAACTTCGTAGCGTCGCTCTGTGGCGCTGGC

Reverse primer with **Xma1** restriction site:

GAGA**CCCGGG**TCAGACGTCCGCGGTTCTCG

The cDNA of all the regulatory subunits were inserted into the pPol_Not1 vector (a generous gift from Dr. Paul Isenring, Université Laval), an oocyte expression vector

containing the T7 promoter (5′ to 3′), the *Xenopus laevis* ß-globin 5′-untranslated region, a multiple cloning site, the *Xenopus laevis* ß-globin 3′-untranslated region, a linearizing site, and polyA and polyC tracts. The cDNA corresponding to the two Na_V1 fragments were inserted in the TOPO vector.

The vectors containing the regulatory subunits were amplified in *Escherichia coli* XL2 Blue (Agilent) and were purified using GenElute HP Plasmid Maxiprep kits (Sigma).

The constructs were linearized with Not1, and T7 RNA polymerase was used to make sense RNA using mMESSAGE mMACHINE T7 kits (Ambion). mRNA was also generated using mMESSAGE mMACHINE kits with a custom DNA featuring a T7 promoter, the $AmNa_V1$ channel sequence, the *Xenopus laevis* ß-globin 3′-untranslated region, and polyA and polyC tracts.

To identify sequence variations, genomic DNA from whole bees was extracted and was sequenced using QIAamp DNA mini kits (Qiagen).

Supplemental methods: oligonucleotide primer sequences for tissue expression analysis

The following primer sequences were used for RT-PCR amplifications in order to visualize the tissue expression pattern of the proteins under investigation:

For $AmNa_V1$

5’-GATCCGATGCTCGAACAAGGGC-3’ (forward primer)

5’-GGGCATGATCATGAGTATGCAGGC-3’ (reverse primer)

For TipE

5’-AAGTGGACCCCGGGATAGTCATCAGC-3’ (forward primer)

5’-CGACCTTCAGCTGATGCCCAAAGC-3’ (reverse primer)

For TEH1

5’-CATCTGCACCACCTCCAGACGC-3’ (forward primer)

5’-ATTTCGCGGGTGAAGTTCTCGGC-3’ (reverse primer)

For TEH2

5’-TAAATTCTACACCTCCCTCTGCCTGC-3’ (forward primer)

5’-CACTGTATGGAGCCCAACGGTTTCGC-3’ (reverse primer)

For TEH3

5’-TCTACCTGACCGTTGCCATCTACAGC-3’ (forward primer)

5’-TTCGACACTCGTCCGCTATGCGC-3’ (reverse primer)

For TEH4

5’-GAACAAAGCGAATGGAAATCTGCCGC-3’ (forward primer)

5’-TAGAGGCGAGAACTGTTGTAGATGC-3’ (reverse primer)

Supplemental methods: Equations

The fraction of channels modified by pyrethroid insecticides was calculated using the following equation:

$$M(\%) = 100 \times \frac{I_{tail}/(E_{tail} - E_{rev})}{I_{dep}/\left(E_{dep} - E_{rev}\right)}$$

where I_{tail} is the maximal amplitude of the tail current measured in the 170s following the last conditioning pulse (the first 5ms are excluded to avoid the inclusion of a capacitive component in the current measured), E_{tail} is the membrane potential at which the tail current was measured, E_{rev} is the reversal potential of sodium ions as calculated using an I-V curve, and I_{dep} is the peak current observed in response to a depolarization at the E_{dep} potential.

The dose-response curves obtained from these calculations were fitted to a Hill curve using the following equation:

$$M = \frac{M_{Max}}{1 + (EC_{50}/dose)^h}$$

where h is the Hill coefficient, M_{max} is the maximal fraction of channels affected, EC_{50} is the half-maximal effective concentration, and dose is the concentration at which the observation was made.

The voltage-dependence of activation was fitted to the following Boltzmann equation:

$$\frac{G_{(V)}}{G_{Max}} = \frac{1}{1 + exp^{\frac{(V - V_{1/2})}{k}}}$$

while the voltage-dependence of inactivation was fitted to the following equation:

$$\frac{I_{(V)}}{I_{Max}} = \frac{1}{1 + exp^{\frac{(V - V_{1/2})}{k}}}$$

where $V_{1/2}$ is the half-maximal voltage of activation (or inactivation), G is the conductance, I is the current measured at a given voltage (V), and k is the slope factor (in mV).

Current decay and tail current kinetics were fitted to the following equation when a single exponential was used:

$$y = y_o + A\left(1 - exp\left(\frac{-x}{\tau}\right)\right)$$

and to the following equation when double exponential was used:

$$y = y_o + A_1\left(1 - exp\left(\frac{-x}{\tau_1}\right)\right) + A_2\left(1 - exp\left(\frac{-x}{\tau_2}\right)\right)$$

where y_0 is the initial value, A is the weight of the exponential, and τ is the time constant of the exponential.

All fits were performed using the Levenberg-Marquardt algorithm.

Supplemental tables:

Table S1: Correspondence of residues in the honeybee Na_V1 sequence with known kdr-linked mutations

Table S2: Residues implicated in the formation of the pyrethroid binding site in the honeybee's Na_V1 channel according to the participating residues in *Aedes aegypti*.

Supplemental figure legends:

Figure S1: Sequence alignment of the homologous domains of the honeybee Na_V1 channel. **(A)** The homologous domains of AmNa$_V$1 are aligned with the sequences of the homologous domains from various mammalian sodium channels. The crystallized bacterian sodium channel Na$_v$Ab was used as reference to identify the 6 transmembrane segments comprised in each domain. Conserved residues are annotated with "*", ":" or "." according to the ClustalW consensus representation. The highly conserved amino acids which form the GCTC, the selectivity filter (SF) and the conserved positive charges on S4 are highlighted. Conserved negative, positive, aromatic and uncharged residues are shown in red, blue, green and grey respectively. **(B)** Sequence alignment of the putative hydrophobic plug for AmNa$_V$1. The IFMT motif and its MFMT counterpart are shown in bold. The putative phosphorylation site identified with pBlast is highlighted in orange. **(C)** 2-D representation of the motifs found in the honeybee Na_V1 channel.

Figure S2: Sequence variants of the investigated proteins and chromosomal organization of the AmNa$_V$1 regulatory subunits. **(A-B)** Sequence variants found for the AmNa$_V$1 channel and its regulatory subunits. Green annotations correspond to insertions whereas the red annotation represents a deletion. Red and green circles represent alterations which are the result of genomic variations and RNA edition respectively.

Figure S3: Sequence alignment the TipE and TEH regulatory subunits. **(A)** The regulatory subunits are aligned with the $hK_{Ca}\beta2$ protein which modulates the biophysical properties of the mammalian K_{Ca} channels. Conserved residues are annotated with "*", ":" or "." according to the ClustalW consensus representation. The transmembrane segments identified with SOSUI are shown in bold. The regions where most transmembrane segments are found are framed. The cysteines thought to be important for the formation of disulphide bridges in $hK_{Ca}\beta2$ are highlighted in grey. Highly conserved cysteines found in the drosophila's and honeybee's Na_V1 regulatory subunits are highlighted in orange. **(B)** Alignment of the two EGF-like domains found in TEH3 and TEH4. Cysteines involved in disulphide bonds are highlighted in grey. Residues in the first EGF-like domain potentially involved in calcium binding are shown in bold. **(C)** 2-D representation of the regulatory subunits.

Figure S4: $AmNa_V1$ single-channel conductance**. (A)** Single-channel traces from cell-attached oocytes patch are illustrated for -10, -20, -30 and -40 mV. Arrows indicate the onset of patch depolarization from −100 mV to the indicated voltage for 150 ms. (**B)** All-points histogram of the single-channel current amplitudes at -30 mV. The smooth curve is a Gaussian fit with amplitude peaks at 1.77 pA. **(C)** Plot of the current–voltage relationship ($n \geq 7$). The straight line is a linear regression yielding a unitary conductance of 24.5 ± 0.2 pS. Data are presented as mean ± SEM.

Figure S5: Effect of TTX on $AmNa_V1$. **(A)** The channel exhibited a reversible, dose-dependent block by TTX. Using oocytes expressing $AmNa_V1$ and TipE, the fraction of channels blocked at different concentrations of TTX were recorded (n = 5). The data was then fitted with a Hill curve in order to extract the Hill coefficient (h = 1,6) and the half maximal inhibitory concentration (IC_{50} = 0,7 nM).

	Original Amino Acid	Numbering *Musca Domestica*	Mutated Amino acid	Associated mutations	Species	*Apis Mellifera* Amino Acid	*Apis Mellifera* Numbering
	I	254	N	None	Drosophila melanogaster	I	264
	V	410	A, G, L, M	None E435K+C785R	Helicoverpa zea, Cimex luctularis	V	419
	E	435	K	V410M+C785R, C785R+L1014F	Blattella germanica	E	444
*	E	485	K	C785R + L1014F	Blattella germanica	D	489
*	C	785	R	E485K+ L1014F, L1014F	Blattella germanica	C	789
	M	827	I, T	None, T929I, L932F, L1014F	Pediculus humanus capitis, Pediculus humanus corporis	M	831
*	M	918	I, L, T, V	None, M918L+L925I, M918I+L1014F, M918T+L1014F	at least 11 different	M	922
	L	925	I	None	Bemisia tabaci, Cimex luctularis, Trialeurodes vaporariorum, Rhipicephalus microplus	L	929
*	T	929	C, I, N, V	None, M827I, L932F, L1014F, L1014F+A1060T+P1879S	at least 13 different	T	933
	L	932	F	M827I, M827I+T929I	Pediculus humanus capitis, Pediculus humanus corporis	L	936
	G, C	933	V, A	None	Rhipicephalus microplus	C	937
	I	936	V	None	Helicoverpa zea	I	940
	Q	945	R	None	Lepeophtheirus salmonis	Q	949
*	F	979	S	L1014F	Myzus persicae	F	983
	S	989	P	V1016G	Aedes aegypti	S	993
*	V	1010	L	L1014S	Anopheles culicifacies, Anopheles sinensis	V	1014
	I	1011	M, V	None	Aedes aegypti, Anopheles sinensis	I	1015
	N	1013	S	None	Anopheles sinensis	N	1017
	L	1014	C, F, H, S, W	Identified with *	At least 20 different	L	1018
	V	1016	G, I	None, D1763Y	Aedes aegypti	V	1020
	F	1020	C	None	Blattella germanica, Plutella xylostella	F	1024
	L	1024	V	None	Tetranychus urticae	L	1028
*	A	1060	T	T929I+L1014F+P1879S, L1014F+N1575Y, L1014F+P1879S, P1879S	Plutella xylostella	A	1099
	A	1215	D	None	Tetranychus urticae	Poor alignment	
	A	1410	V	None	Drosophila melanogaster	A	1409
	A	1494	V	None	Drosophila melanogaster	A	1493
	M	1524	I	None	Drosophila melanogaster	M	1523
	F	1528	L	L1596P+I1752V+M1823I	Varroa destructor	F	1527
	F	1534	C	None	Aedes aegypti, Aedes albopictus	F	1533
	F	1538	I	None	Rhipicephalus microplus, Tetranychus cinnabarinus, Tetranychus urticae	F	1538
	D	1549	V	E1553G	Helicoverpa armigera, Heliothis virescens	D	1548
	E	1553	G	D1549V	Helicoverpa armigera, Heliothis virescens	E	1552
*	N	1575	Y	L1014F	Anopheles gambiae	N	1574
	L	1596	P	None, F1528L+I1752V+M1823I	Varroa mite	P	1595
	I	1752	V	F1528L+L1596P+M1823I	Varroa mite	I	1751
	D	1763	Y	V1016G	Aedes aegypti	D	1762
	M	1823	I	F1528L+L1596P+I1752V	Varroa destructor	I	1822
*	P	1879	S	T929I+L1014F+A1060T, A1060T	Plutella xylostella	P	1878

Aedes aegypti				Apis Mellifera		
Residue label	Original amino acid	Numbering	Mutated amino acid	Numbering	Original amino acid	Conserved
1k7	I	259	A	258	I	Yes
1k11	V	263	A	262	V	Yes
1o6	L	270	I	269	L	Yes
1o10	I	274	C	273	I	Yes
1o13	T	277	W	266	T	Yes
1i18	L	281	G	280	L	Yes
2i13	I	990	M	1015	I	Yes
2i16	L	993	F	1018	L	Yes

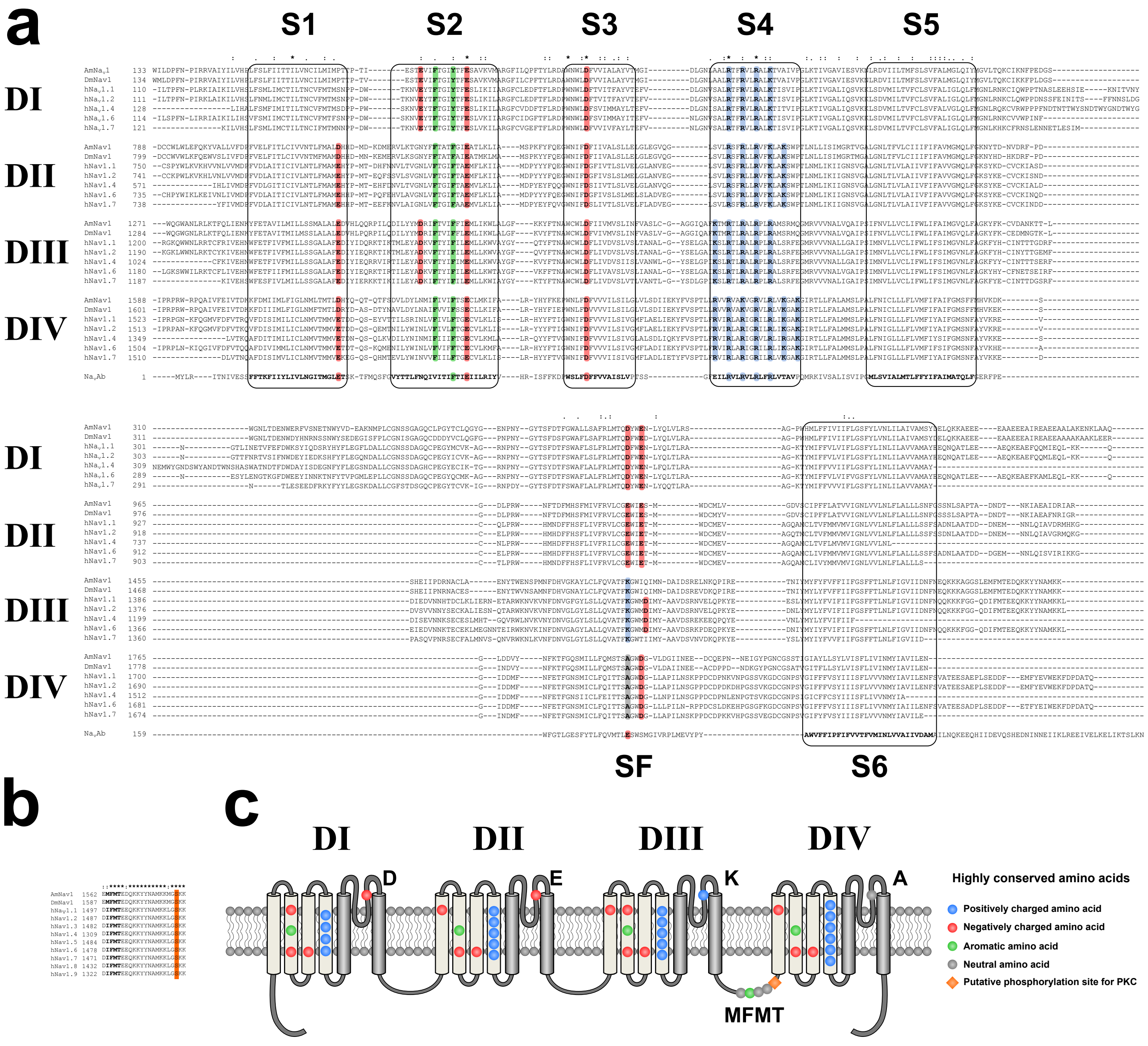

a
S1
S2
S3
S4
S5
DI
DII
DIII
DIV
SF
S6
b
c
DI
DII
DIII
DIV
D
E
K
A
MFMT
Highly conserved amino acids
Positively charged amino acid
Negatively charged amino acid
Aromatic amino acid
Neutral amino acid
Putative phosphorylation site for PKC

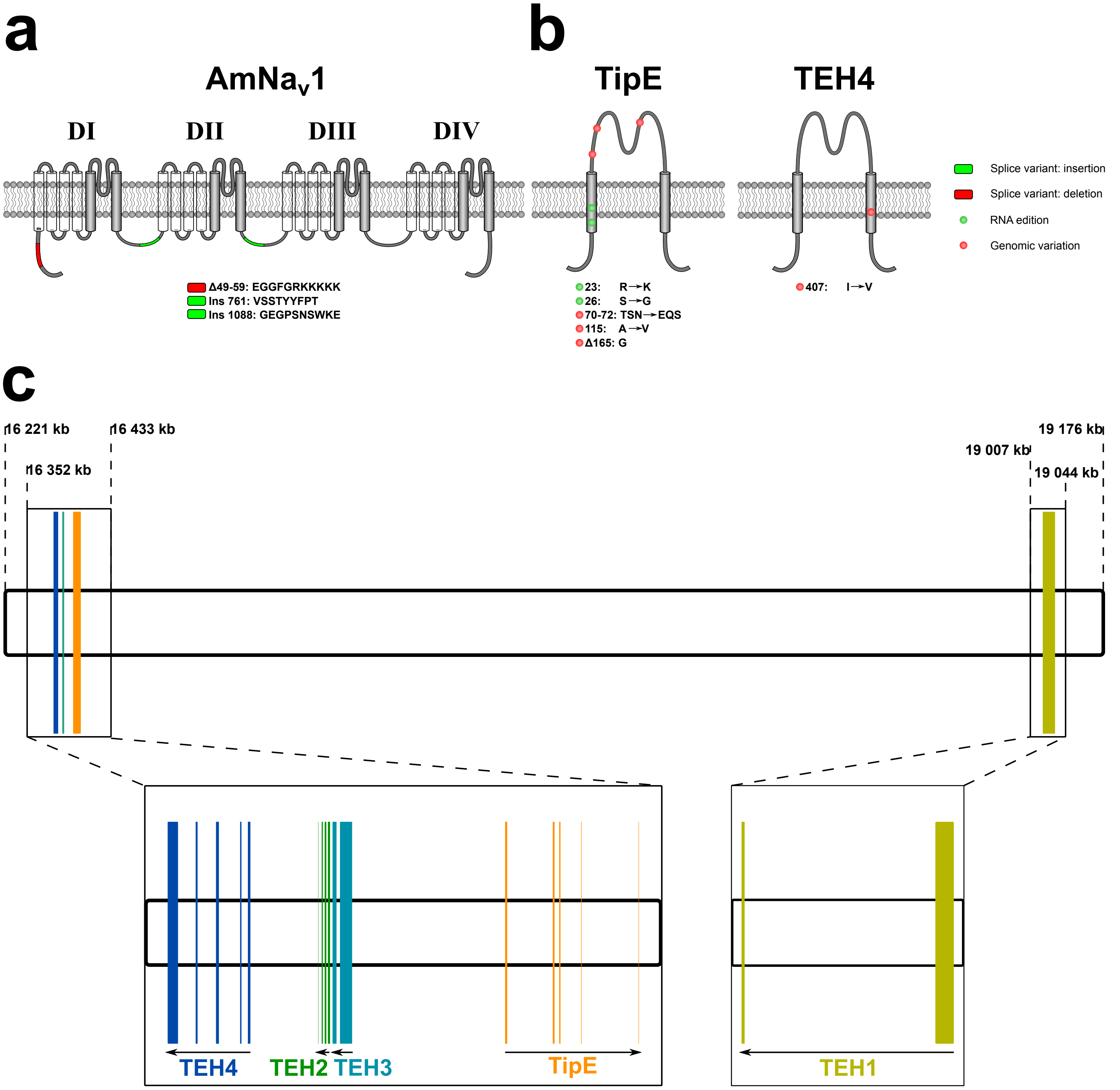
a
AmNav1
DI
DII
DIII
DIV
Δ49-59: EGGFGRKKKKK
Ins 761: VSSTYYFPT
Ins 1088: GEGPSNSWKE
b
TipE
TEH4
Splice variant: insertion
Splice variant: deletion
RNA edition
Genomic variation
23: R→K
26: S→G
70-72: TSN→EQS
115: A→V
Δ165: G
407: I→V
c
16 221 kb
16 433 kb
16 352 kb
19 176 kb
19 007 kb
19 044 kb
TEH4
TEH2
TEH3
TipE
TEH1

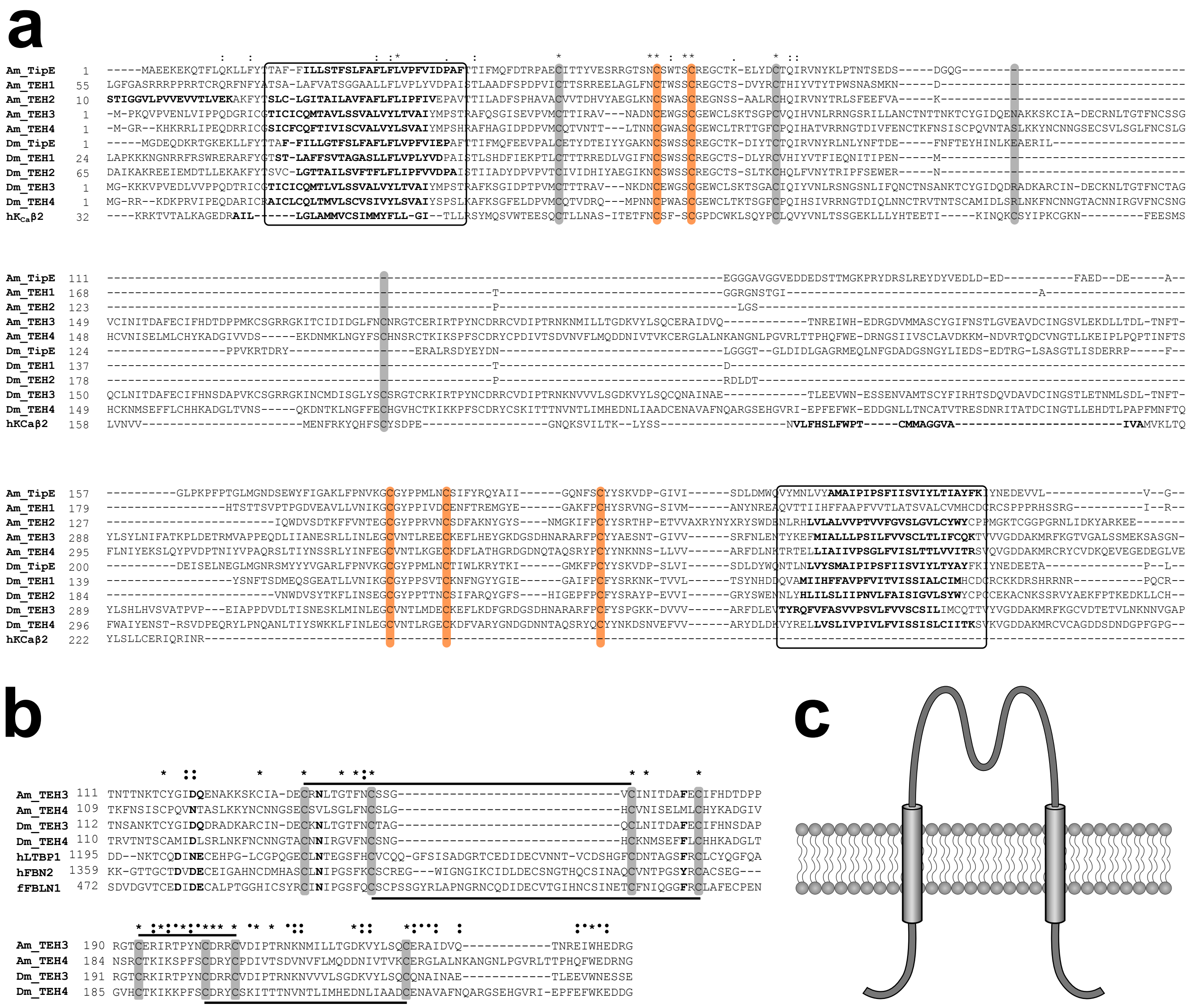
a
Am_TipE
Am_TEH1
Am_TEH2
Am_TEH3
Am_TEH4
Dm_TipE
Dm_TEH1
Dm_TEH2
Dm_TEH3
Dm_TEH4
hKCaβ2
b
hLTBP1
hFBN2
fFBLN1
c

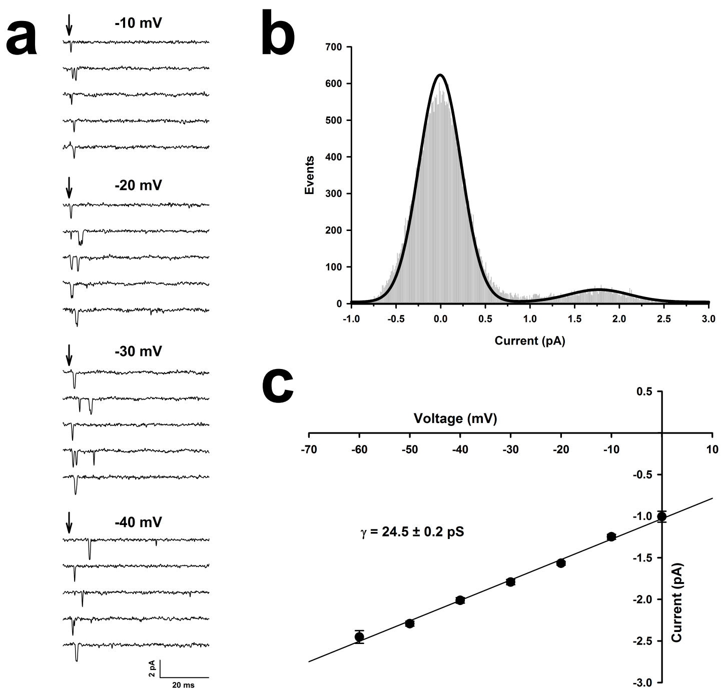

a
-10 mV
-20 mV
-30 mV
-40 mV
2 pA
20 ms
b
Events
Current (pA)
c
Voltage (mV)
γ = 24.5 ± 0.2 pS
Current (pA)

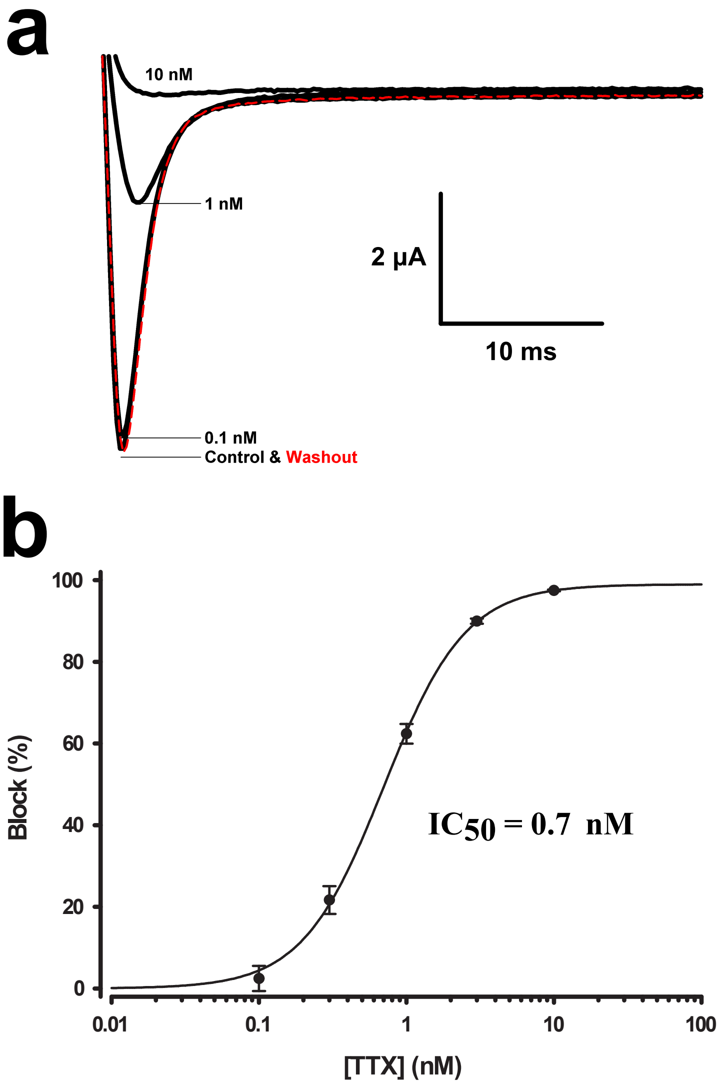

a
10 nM
1 nM
2 µA
10 ms
0.1 nM
Control & Washout
b
100
80
60
40
20
0
Block (%)
IC50 = 0.7 nM
0.01
0.1
1
10
100
[TTX] (nM)
